# Diverse database and machine learning model to narrow the generalization gap in RNA structure prediction

**DOI:** 10.1126/sciadv.adz4967

**Published:** 2026-02-25

**Authors:** Albéric A. de Lajarte, Yves J. Martin des Taillades, Justin Aruda, Pierre Bongrand, Federico Fuchs Wightman, Dragui Salazar, Matthew F. Allan, Colin Kalicki, Casper L’Esperance-Kerckhoff, Alex Kashi, Fabrice Jossinet, Silvi Rouskin

**Affiliations:** ^1^ Department of Microbiology, Harvard Medical School, Boston, MA, USA.; ^2^ Department of Biochemistry, Stanford University, Stanford, CA, USA.; ^3^ Faculty of Life Sciences, University of Strasbourg, Strasbourg, France.

## Abstract

Understanding macromolecular structures of proteins and nucleic acids is critical for discerning their functions and biological roles. Advanced techniques—crystallography, nuclear magnetic resonance, and cryo–electron microscopy—have facilitated the determination of more than 180,000 protein structures, all cataloged in the Protein Data Bank. This comprehensive repository has been pivotal in developing deep learning algorithms for predicting protein structures directly from sequences. In contrast, RNA structure prediction has lagged and suffers from a scarcity of structural data. Here, we present the secondary structure models of 1098 primary microRNAs and 1456 human messenger RNA regions determined through chemical probing. We develop a deep learning architecture inspired from the Evoformer model of Alphafold and traditional architectures for secondary structure prediction. This model, eFold, was trained on our newly generated database and more than 300,000 secondary structures across multiple sources. We benchmark eFold on two challenging test sets of long and diverse RNA structures and show that our dataset and architecture contribute to increasing the prediction performance, compared to similar state-of-the-art methods. Together, our results reveal that merely expanding the database size is insufficient for generalization across families, whereas incorporating a greater diversity and complexity of RNA structures allows for enhanced model performance.

## Teaser

Length matters—secondary structures of long, diverse RNAs in RNAndria and deep learning model eFold improve prediction accuracy.

## INTRODUCTION

The structural integrity of RNA is often critical to its function, playing a vital role in how RNA molecules interact within the cell. While mRNA’s primary sequence is decoded in function (e.g., translation), RNA harbors a second layer of information—its ability to form secondary structures. These structures can conceal or expose crucial regulatory binding sites for proteins, microRNAs, and other RNAs (*1*–*4*). While x-ray crystallography, nuclear magnetic resonance (NMR), and cryo–electron microscopy (cryo-EM) are adept at elucidating RNA’s tertiary structure, their applicability is limited for long RNA sequences [>200 nucleotides (nt)]. Moreover, it is plausible that many long RNAs, apart from specialized ones such as ribosomal RNAs (rRNAs), may not adopt stable tertiary structures, further complicating their studies with conventional methods. As a result, the focus shifts to RNA’s secondary structure, which is an intrinsic characteristic of all RNA molecules. To predict this secondary structure, dynamic programming algorithms are frequently used, identifying the conformation with the lowest energy based on the Turner nearest-neighbor rules (*5*, *6*), a set of experimental parameters for the energy of different base pairs in the context of diverse structure motifs.

Although widely used, these algorithms have their limitations, typically confined to canonical base pairs and structures and often unable to predict more complex interactions such as noncanonical base pairs or pseudoknots. A contrasting approach is to learn RNA structure models directly from data. In this vein, recent advancements have seen the emergence of numerous deep learning methods (*7*–*9*). This methodology makes minimal assumptions about the thermodynamic rules governing RNA structure and, with adequate data, could capture more complex motif types. Nonetheless, a big challenge in deep learning is ensuring a sufficiently diverse and high-quality dataset. A training set lacking in diversity results in poor performance in out-of-domain contexts. This problem is exacerbated in RNA studies because of the unique sequences and structures of different RNA families, leading to many distinct domains. For instance, Flamm *et al.* (*10*) demonstrated how performance markedly declines when algorithms are trained on a specific set of families or sequence lengths and then tested on different ones. Similarly, Bugnon *et al.* (*11*) examined this effect, observing diminished performance when applying published algorithms to very long noncoding RNA (ncRNA) and mRNA. Szikszai *et al.* (*12*) also highlighted the risks of using test sets composed of similar families and advocated for the curation of interfamily test sets.

There are a few databases commonly used by all deep learning methods. The bpRNA database (*13*) consists of more than 100,000 sequences and structures aggregated from multiple sources including Protein Data Bank (PDB) and the RNA families database (Rfam). The PDB database (*14*) includes 1790 RNA tertiary structures as of December 2023. ArchiveII (*15*) and RNAStrAlign (*16*) contain 3975 and 30,451 sequences, respectively. In addition, a substantial dataset of 806,000 sequences, together with chemical probing data for dimethyl sulfate (DMS) and selective 2′-hydroxyl acylation analyzed by primer extension (SHAPE) and data-derived structure models, was recently released for the Ribonanza competition (*17*). Nevertheless, many of these datasets require extensive filtering to eliminate redundant and low-quality sequences. For example, bpRNA90 is a curated subset of bpRNA featuring 28,000 nonredundant sequences, which is 27% of the original database. In addition, these databases tend to be biased toward a limited number of short ncRNA families, which may adversely affect the generalization capabilities of commonly used algorithms.

We examine the generalization ability of four widely used secondary structure algorithms, each using different methodologies. RNAStructure Fold (*18*) is a classic model that uses the Nearest Neighbors parameters and dynamic programming to determine the minimum free energy of a structure. EternaFold (*19*), adapted from ContraFold (*20*), uses context-free grammar to learn the probability of different motifs and structures. MXFold2 (*8*), a deep learning model, predicts the probability of all potential base pairs and conformations as multiple base-pairing matrices. Both EternaFold and MXFold2 are hybrid approaches, leveraging dynamic programming to determine the minimum free energy structure from the predicted energy motifs. UFold (*7*), on the other hand, predicts a base-pairing matrix from the sequence using only a neural network (“end-to-end model”) outfitted with a minor postprocessing step for conversion into a valid structure. We benchmark these algorithms on a set of challenging sequences. The results indicate poor generalization capabilities. This finding led us to develop a new database of relevant and diverse sequences to mitigate the generalization gap.

The state-of-the-art method for high-throughput RNA structure prediction involves probing the RNA molecule with reagents that measure the probability of each nucleotide being paired or unpaired. The two most frequently used methods are DMS-MaPseq and SHAPE. In DMS-MaPseq, DMS reacts at the Watson-Crick face of adenine and cytosine, while SHAPE identifies the most flexible parts of the RNA backbone, typically where unpaired bases are found. Both methods induce mutations in the sequence during reverse transcription, which can be detected through high-throughput next-generation sequencing. Analyzing a multitude of sequencing reads provides a mutation fraction per nucleotide that, after normalization, is interpreted as a pairing probability. These data, when added as a pseudo-energy term to classical algorithms, substantially enhance the accuracy of the predicted structure (*21*, *22*). Here, we used the DMS-MaPseq method to probe and model the structure of a variety of RNAs from families underrepresented in PDB. Our efforts aim to improve generalization across different RNA domains. The data collected, covering biologically important sequences such as the 3′ ends of mRNAs and primary microRNAs (pri-miRNAs) of the human genome, are now accessible in our online database (https://rnandria.org/). Using these new data along with existing RNA structure databases, we trained eFold, based on the Evoformer from Alphafold (*23*) and convolutional neural networks (CNNs). We curate a set of challenging test structures and demonstrate enhanced generalization capabilities compared to state-of-the-art deep learning models.

## RESULTS

### Secondary structure algorithms do not generalize across different types of RNAs

**Fig. 1.**
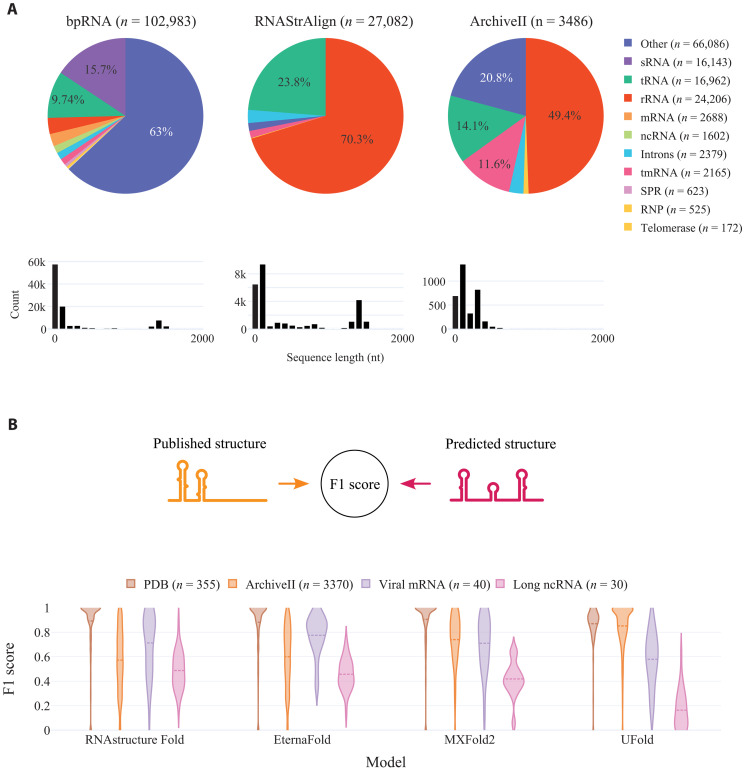
Performance of state-of-the art algorithms on diverse RNA domains. (**A**) RNA types and sequence length distribution of bpRNA, RNAStrAlign, and ArchiveII datasets. (**B**) Violin plot illustrating the distribution of F1 scores comparing the predicted structure model by each algorithm against a published structure model across four distinct test sets: PDB, ArchiveII, Viral mRNAs, and Long ncRNAs. We group the results per model to highlight the effect of testing on out-of-domains datasets. The F1 score, calculated as the harmonic mean of precision and sensitivity, spans from 0 to 1.

Current secondary structure predictions rely on the same databases for their training sets. A notable issue is the lack of diversity in these datasets, which are predominantly biased toward short ncRNAs. Rfam (*24*) is a database of all the known RNA families and clans, counting 146 clans to date, and consistently adding new ones. However, only a few clans such as tRNA, rRNA, and small RNA (sRNA) are substantially represented in commonly used databases, and most sequences are typically less than 200 nt long (Fig. 1A and fig. S1, A to D). This bias allows deep learning models to excel within these family domains, but their performance substantially diminishes outside of them (*10*–*12*).

To evaluate performance beyond common testing sets, we curated four test datasets from published RNA structures. The first dataset comprises RNA structures from the PDB, consisting of 356 sequences obtained by excluding entries with multiple chains, such as RNA-RNA, RNA-DNA, or RNA-protein complexes, resulting in a collection of predominantly short ncRNAs. Our second dataset, ArchiveII, is a commonly used test set in previous studies (*7*–*9*). The final two test sets aim to evaluate performance across new families and various sequence lengths. The long ncRNA (lncRNA) dataset (Long ncRNA), curated by Bugnon *et al.* (*11*), includes 30 long RNA sequences ranging from 1000 to 2000 nt, with structures exceeding 2000 nt segmented into smaller modular structures, as detailed in Methods. The fourth dataset consists of 40 sequences derived from multiple published viral genomes. Given that the initial viral RNA sequences exceed 10,000 nt, we segmented them into modular structures. Our segmentation approach aimed to ensure that the segmented structure aligns with the original structure within the longer molecule. Essentially, we divided the lengthy structure into smaller, self-consistent modules, typically ranging from 150 to 300 nt for practicality. We ensure that those modular structures conserve structural domains by only keeping the ones showing high agreement with the original structure (F1 score > 0.8). The full curation process for these datasets is described in Methods. All test sets present unique challenges (bias, protein-assisted folding, and structure model errors), making it difficult to establish a definitive “gold standard” structure because of the sensitivity of RNA folding to experimental conditions and the presence of alternative structures. However, collectively, these test sets offer valuable insights for assessing variations in performance, particularly the capability of machine learning (ML) models to align with structure models derived from chemical probing data.

We evaluated four widely used algorithms on these curated test sets: RNAstructure Fold, EternaFold, MXFold2, and UFold, by calculating the F1 score distribution between the predicted and published structure models (Fig. 1B). The F1 score, a commonly used metric in classification, balances precision and sensitivity in detecting correct base pairs, where a score of 1.0 signifies identical structures. All algorithms exhibited remarkable efficacy on the PDB dataset, typically achieving an F1 score around 0.9 (Fig. 1B and Table 1). However, their performances notably declined on the more challenging test sets. For the viral structures, the average F1 score was around 0.7, and it was even lower for the lncRNAs, with an average score of approximately 0.45 for RNAstructure Fold, EternaFold, and MXFold2 (Fig. 1B and Table 1). Among these, UFold demonstrated the weakest performance, especially in predicting base pairs in lncRNAs. This variation in performance across different test sets suggests that ML-based algorithms are primarily developed or trained for RNA types frequently encountered in the PDB and ArchiveII datasets, with limited exposure to newer RNA domains. It is notable that nearly half of the sequences in the ArchiveII test set exhibit more than 80% similarity (fig. S1C) with bpRNA and RNAStrAlign, two databases commonly used for training. UFold’s lack of generalization is particularly pronounced when compared with MXFold2, a hybrid algorithm, as UFold is a fully end-to-end algorithm without built-in assumptions on base-pairing rules or RNA thermodynamics. To mitigate these limitations, we compiled a new dataset aimed at training ML-based algorithms to improve their adaptability and performance across diverse RNA domains.

**Table 1.** Performance of state-of-the art algorithms on diverse RNA types. Average precision, recall, and F1 score are reported by first computing each metric for every predicted structure, then averaging across all structures within each test set. Average precision, recall, and F1 score between the predicted structure model of each algorithm against a published structure model across four distinct test sets: PDB, ArchiveII, Viral mRNAs, and Long ncRNAs. The best model per test (column) is in bold.

Dataset	PDB			ArchiveII			Viral mRNA			Long ncRNA		
	Precision	Recall	F1	Precision	Recall	F1	Precision	Recall	F1	Precision	Recall	F1
**Model**												
RNAstructure Fold	0.90	0.91	0.89	0.55	0.60	0.57	0.69	0.74	0.71	**0.46**	**0.52**	**0.49**
EternaFold	0.88	0.91	0.88	0.57	0.64	0.60	**0.75**	**0.81**	**0.77**	0.45	0.47	0.46
MXFold2	**0.91**	0.93	**0.90**	0.73	0.76	0.74	0.70	0.72	0.71	0.41	0.43	0.42
UFold	0.81	**0.97**	0.87	**0.83**	**0.89**	**0.85**	0.58	0.59	0.58	0.22	0.14	0.16

### A new database of biologically relevant RNAs to bridge the generalization gap

**Fig. 2.**
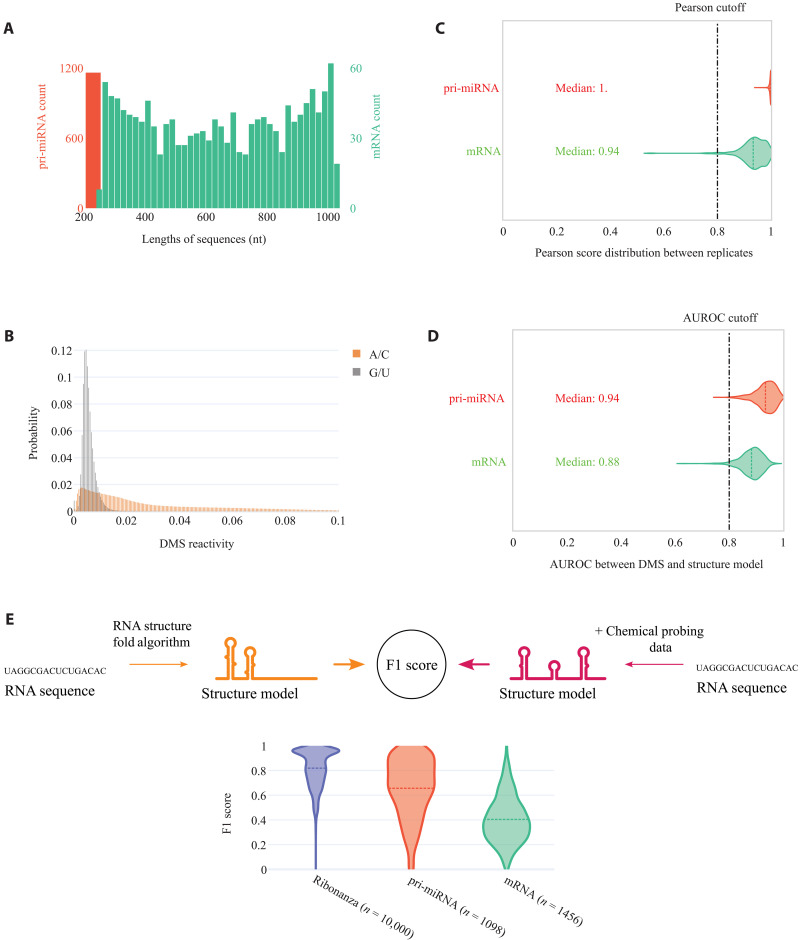
Data quality and complexity of RNAndria database. (**A**) Sequence length distribution for the pri-miRNA dataset (left scale, red) and mRNA dataset (right scale, teal). (**B**) Distribution of non-normalized DMS reactivity for the pri-miRNA and mRNA datasets. The nonreactive G/U bases are highlighted in gray with a mean reactivity of 0.006, while the reactive A/C bases are in orange with a mean reactivity of 0.03. (**C**) Distribution of Pearson correlation scores between DMS signal replicates. The dotted line corresponds to the threshold of 0.8 used for filtering. (**D**) Distribution of AUROC between the structure models and DMS signals. The dotted line corresponds to the threshold of 0.8 used for filtering. (**E**) Distribution of F1 score between structures predicted by RNAstructure Fold from sequence alone compared to RNAstructure Fold with chemical probing constraints, for Ribonanza, pri-miRNA, and human mRNA region (mRNA) dataset.

To address the generalization gap in RNA secondary structure prediction, we used DMS-MaPseq (*21*) to probe 4550 sequences from the 3′ end of human mRNAs and 1292 pri-miRNA sequences, which include the precursor hairpin and flanking regions (Methods). Together, the distribution of lengths ranges from 200 to 1000 nt (Fig. 2A). Previous studies have shown that adding chemical probing constraints to RNAStructure yields models that are 90 to 100% identical to models obtained through crystallography, cryo-EM, or phylogenetic covariation (*21*, *25*). To ensure high-quality DMS signal, we used a stringent coverage cutoff of at least 3000 reads per base (Methods). The median signal for the DMS-reactive bases, adenine and cytosine (AC), over uracil and guanine (GU) was 5.3-fold, with a two phase distribution between reactive and nonreactive bases (Fig. 2B). DMS reactivity for each RNA was then used as constraints in RNAstructure Fold’s algorithm to model the secondary structure. We also generated the structure models using the DMS reactivity as constraint with ShapeKnots and compared it with RNAstructure Fold. Those two methods give very similar structure on the pri-miRNA dataset, with an average F1 score of 0.98 and 72% of the dataset having exactly the same structure (F1 score = 1). Although the constraint parameters have undergone optimization (*25*), it is important to note that models derived from these data might not always align with the actual data. This discrepancy occurs because RNAstructure Fold produces an output regardless of its ability to find a suitable match for the data, a point further explored in the discussion on Area Under the Receiver Operating Characteristic curve (AUROC) below.

We assessed the quality of the final dataset in several ways. More than 70% of sequences underwent dual independent probing. The correlation between the DMS signal replicates is depicted in Fig. 2C. The reproducibility of the signals is highlighted by a high mean Pearson correlation between DMS replicates of 0.95 for the mRNA and 0.99 for the pri-miRNA. To evaluate the agreement of the derived structure models with the DMS data, we used AUROC. The ROC curve plots the trade-off between precision (true-positive rate) and sensitivity (false-positive rate) of a binary classifier, as it differentiates between paired and unpaired bases at varying thresholds of DMS signal intensity. At each threshold, it computes the true-positive rate and false-positive rate in comparison to a given structure model. An AUROC score of 1.0 signifies the existence of a specific threshold where all DMS-reactive positions correctly align with loop regions and all DMS-unreactive positions with stem regions, demonstrating perfect agreement with the observed data. Although a high AUROC does not validate the model (there could exist two or more conformations that perfectly fit the data), a low AUROC indicates that the model is incorrect, either because of the presence of alternative structures or because of poor data quality. “Gold standard” structures determined by crystallography or NMR have high AUROCs (>0.8) (*26*). We present the AUROC values for each structure in Fig. 2C and our newly established database, RNAndria, and exclude any structure with an AUROC below 0.8 (Methods).

We highlight the importance of integrating DMS signals into our structure models by comparing the performance of RNAstructure Fold with and without DMS constraints. Our analysis reveals a large variation in model performance for mRNA and pri-miRNA datasets—F1 scores range from 0.3 to 0.7—when relying solely on sequence data versus when DMS constraints are included (Fig. 2D). This discrepancy in F1 scores aligns with the marked improvements in RNAstructure Fold’s accuracy for rRNA, which jumps from ~50 to 90% with the incorporation of chemical probing data (*21*). The notably low F1 scores for RNAstructure Fold on the RNAndria dataset indicate the challenge of predicting complex structures in the absence of probing data. In contrast, models derived from Ribonanza (*17*), the only other extensive chemical probing dataset, show minimal variance with or without chemical constraints (Fig. 2D). This suggests that the sequences in Ribonanza fold into motifs that are more predictable. The stark difference highlights the distinct value of our dataset in enhancing model training, enabling better generalization across a broad spectrum of RNA structural motifs.

**Fig. 3.**
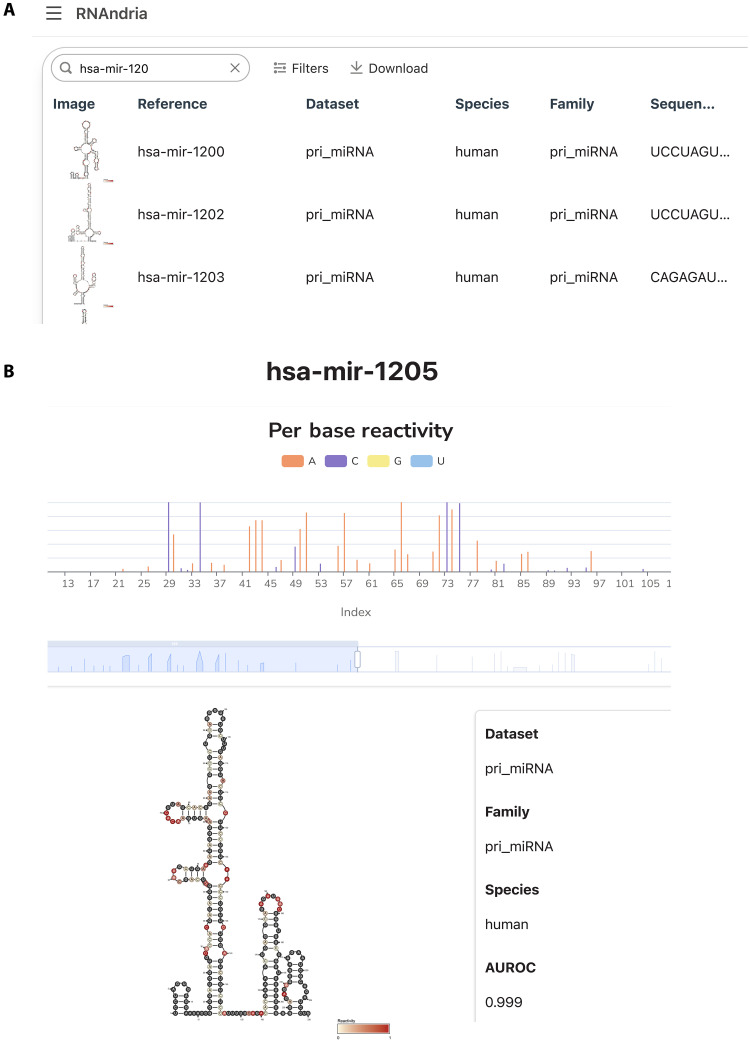
RNAndria database architecture. (**A**) Main page of the online database RNAndria, showing the browsing, filtering, searching, and download features. (**B**) Subpage example for one sequence of the pri-miRNA hsa-mir-1205, showing its secondary structure, DMS reactivity, and metadata.

RNAndria postfiltering for high read depth includes 1456 mRNA and 1098 pri-miRNA structures. This dataset is available in an online database, https://rnandria.org/, which allows users to search and filter by data features including species, condition, dataset, structural motifs, and more. Researchers can then visualize and download their data of interest. We included visualization of both the secondary structure and DMS signal (Fig. 3). The secondary structures, along with their DMS reactivity per base, were performed using RNArtist (Methods), a tool suited for representing long and complex RNA structures. We intend to continue improving this database in the future, by increasing its size with new datasets and refining our methods to improve the structural models.

### An ML architecture for secondary structure prediction

**Fig. 4.**
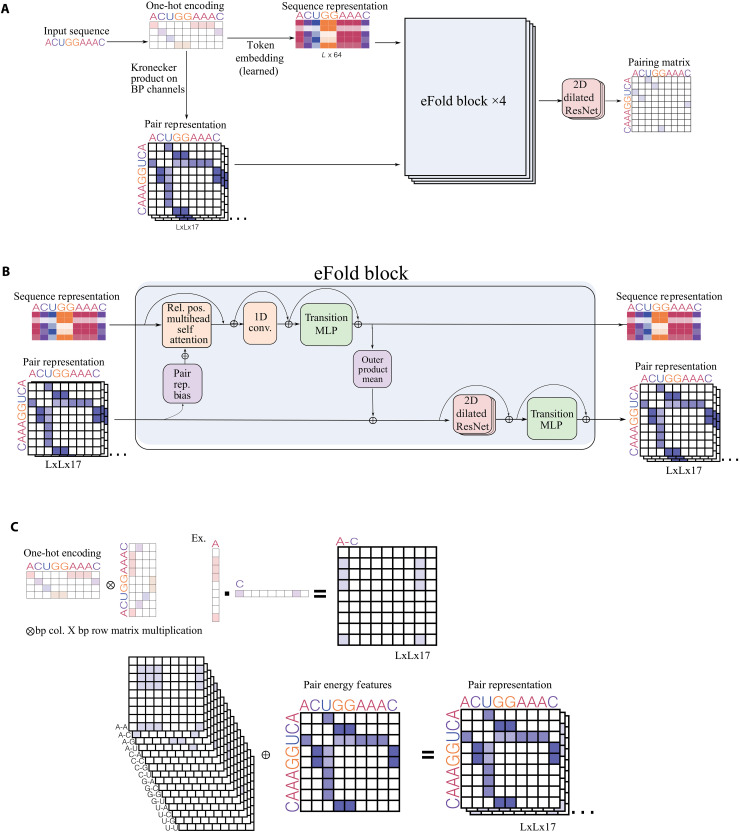
eFold architecture. (**A**) High-level schematic of the eFold architecture, with the two input representations and the output pairing matrix. (**B**) Low-level view of one eFold block, showing the sequence and pair representation channels, as well as the interchannel connections. The sequence representation is only used within the Evoformer blocks to share information with the pair representation and is then discarded. (**C**) Details on the computation of the input pair representation from sequence. Similar to UFold, we use 16 binary matrices to represent all base interactions and one channel to represent the energy of A-U, G-C, and G-U pairs.

We next explored a neural network architecture for RNA structure prediction adapted from the Evoformer module of Alphafold (*23*). This model, which we called eFold, consists of four blocks depicted in Fig. 4. The core design of each eFold block contains two channels: One channel processes the sequence representation through self-attention layers, and the other handles pairwise representation via ResNet convolutional layers. Specialized connections between layers in each channel facilitate effective information exchange. CNNs have been heavily used in many deep learning structure prediction models and are thus integral to our architecture. Their suitability for handling image-like representations of pairing matrices, along with their computational efficiency, makes them preferable over the graph neural network blocks found in the original Evoformer. However, self-attention layers complement CNNs by learning global context across the sequence without imposed range limitations. This capability could enable the model to identify complex interdependencies and interactions between distant nucleotides, potentially facilitating the selection of globally optimal base-pairing solutions among competing alternatives.

As shown in Fig. 4A, the sequence representation is initialized using a simple table embedding, while the pair representation adopts a method similar to that in the UFold architecture (*7*). This setup encodes the sequence as a binary map representing all 16 possible base pair interactions and includes an additional channel that factors in the energy of different pairs. Following the eFold blocks, only the pair representation is advanced further. It is then processed through a ResNet block, resulting in the generation of the pairing probability matrix. This architecture is designed to balance the strengths of both CNNs and self-attention layers, aiming to enhance the accuracy and efficiency of RNA secondary structure prediction from sequence data.

### Enhanced performance of eFold in predicting the structure of long RNAs

**Fig. 5.**
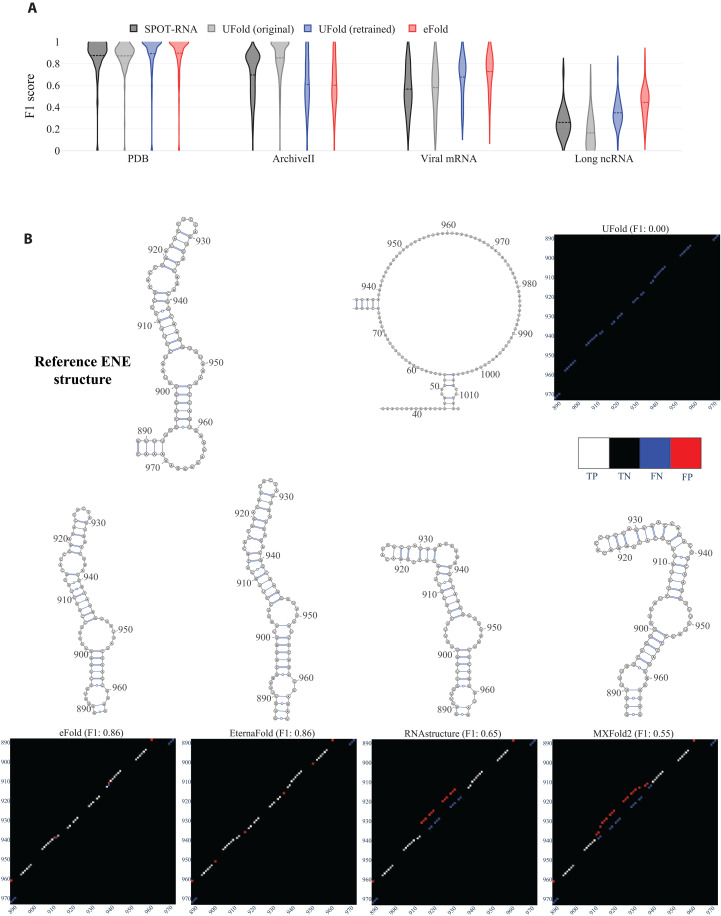
eFold performance. (**A**) F1 score distribution between each model prediction and the ground truth, for the four test sets. eFold in red is pretrained and then fine-tuned on the mRNA and pri-miRNA datasets. In blue is the UFold architecture being trained with the same training recipe and data as eFold in red. We group the results per test set to highlight the difference in performance between models. (**B**) Secondary structure model of the ENE (element for nuclear expression), a functional element within PAN of Kaposi’s sarcoma herpesvirus. The full secondary structure is predicted with each algorithm; then, the ENE is drawn using VARNA, and the predicted and reference base pairing matrix are plotted next to each model, showing the correct base pairs in white [true positive (TP)], the missing base pairs in blue [false negative (FN)] and incorrect base pair predictions in red [false positive (FP)].

Our model underwent a two-phase training process. Initially, we used existing databases, including bpRNA and Ribonanza, to compile a comprehensive pretraining dataset comprising more than 120,000 unique sequences and structures. To ensure data quality, redundant sequences were filtered out using BLAST (*27*), and low-quality structure models were discarded (fig. S2B). In addition, we incorporated 220,000 sequences from RNAcentral and used RNAstructure Fold to generate their structures. This synthetic dataset facilitated our model’s learning of RNAstructure Fold’s basic pairing rules, enabling better generalization across new families and sequence lengths. A summary of the datasets used for pretraining, fine-tuning, and testing is presented in Table 2. The pretraining phase alone permitted our model to surpass the performance of the current best end-to-end model, UFold, on the lncRNA test set, achieving a mean F1 score of 0.40 versus UFold’s 0.16, and SPOT-RNA 0.26 (Fig. 5A). On the viral mRNA test set, just pretraining eFold allows to reach a mean F1 score of 0.67, while UFold reaches 0.58 and SPOT-RNA reaches 0.56.

**Table 2.** Summary of training datasets. Description of each dataset used either as pretraining, fine-tuning, or testing, along with their curation method, source of data, family, and length information.

Training stage	Name on HuggingFace	Source	Method	Number of sequences	Families	10–199 nt	200–499 nt	500–999 nt	1000–1999 nt	>2000 nt
Pretraining	rnacentral_synthetic	Sequences from RNA central	RNAstructure	226,729	All known families	176,486	49,463	780	0	0
Pretraining	ribo500-blast	Ribonanza competition	RNAstructure + DMS and/or SHAPE	46,060	Unlabeled	46,049	11	0	0	0
Pretraining	bpRNA-1 m	bpRNA-1 m	Covariance analysis	66,715	Unlabeled, sRNA, tRNA	48,090	6,167	2,829	9,260	369
Fine-tuning	pri_miRNA	This work	RNAstructure + DMS	1,098	pri-miRNA	0	1,098	0	0	0
Fine-tuning	human_mRNA	This work	RNAstructure + DMS	1,456	mRNA	0	493	882	81	0
Testing	PDB	PDB	NMR, crystallography	356	Short ncRNA	343	6	6	1	0
Testing	viral_fragments	Peer-reviewed literature	RNAstructure + DMS	40	Viral RNA	12	17	11	0	0
Testing	lncRNA	Bugnon *et al. *(*11*)	RNAstructure + DMS	10	Long ncRNA	0	2	1	7	0
Testing	archiveII_filtered	Archive II	Covariance analysis	355	rRNA, tRNA, tmRNA, unlabeled	242	65	43	5	0

In the subsequent phase, we fine-tuned our model with curated mRNA and pri-miRNA datasets, which led to improved mean F1 scores across all test sets, demonstrating the importance of new and diverse data in enhancing model generalization (Fig. 5A and Table 3). The final eFold model outperformed in the two most challenging test sets, registering a mean F1 score of 0.73 on the viral mRNA compared with UFold’s 0.58 and SPOT-RNA’s 0.56, and 0.44 on the lncRNA, against UFold’s 0.16 and SPOT-RNA’s 0.26. A comprehensive benchmark including additional end-to-end secondary structure prediction models (E2EFold, CNNFold, NeuralFold, and RNAFormer) is provided in table S1, while Table 3 and Fig. 5A focus on the best-performing models (SPOT-RNA, UFold, and eFold) for clarity. Figure S3 details how training with each database affects eFold performance, showing that RNAStrAlign and bpRNA contribute minimally to accuracy on the most challenging and biologically relevant test sets (viral mRNAs and lncRNAs), likely due to biases toward particular structure types. In addition, to address potential redundancy from training on RNAstructure predictions, we compared eFold predictions directly to those of RNAstructure and found only moderate correlation (Pearson correlation = 0.67 for viral mRNAs and 0.44 for lncRNAs; fig. S4), indicating that eFold and RNAstructure remain distinct in their predictions.

**Table 3.** Performance of end-to-end models. Average precision, recall, and F1 score between the predicted structure model of NeuralFold, SPOT-RNA, UFold, eFold without fine-tuning, and eFold with fine-tuning against a published structure model, across four distinct test sets: PDB, ArchiveII, Viral mRNAs, and Long ncRNAs. The best model per test (column) is in bold.

Dataset	PDB			ArchiveII			Viral mRNA			Long ncRNA		
	Precision	Recall	F1	Precision	Recall	F1	Precision	Recall	F1	Precision	Recall	F1
**Model**												
SPOT-RNA	0.85	0.92	0.87	0.69	0.73	0.70	0.68	0.50	0.56	0.34	0.21	0.26
UFold (original)	0.81	**0.97**	0.87	**0.83**	**0.89**	**0.85**	0.58	0.59	0.58	0.22	0.14	0.16
UFold (retrained)	**0.90**	0.92	0.89	0.59	0.64	0.61	0.67	0.70	0.67	**0.55**	0.28	0.35
eFold (pretrained)	0.87	0.93	0.89	0.55	0.64	0.58	0.64	0.72	0.67	0.41	0.40	0.40
eFold	0.89	0.93	**0.89**	0.57	0.64	0.60	**0.70**	**0.75**	**0.73**	0.46	**0.43**	**0.44**

The only test set on which UFold exhibited higher accuracy was ArchiveII (Table 3). Notably, we did not remove any sequences from the ArchiveII test set, so it likely contains sequences similar to those present in the training sets of both UFold and SPOT-RNA (see fig. S5). This overlap may have artificially inflated their performance on the ArchiveII test set.

To directly compare the performance of the UFold and eFold architectures independent of their original training datasets, we retrained UFold using the exact same pretraining and fine-tuning procedures as eFold, and with identical datasets. The resulting retrained UFold model (Fig. 5A) still demonstrated lower performance on the two most challenging test sets: On the viral mRNA test set, UFold achieved a mean F1 score of 0.67, compared with 0.73 for eFold; on the lncRNA test set, UFold scored 0.35, while eFold achieved 0.44. Even without fine-tuning, eFold’s performance on the lncRNA test set (F1 score of 0.40) surpassed that of UFold, even when UFold was both trained and fine-tuned on long RNA (F1 score of 0.35; Table 3).

This performance advantage can likely be attributed to eFold’s architecture, which combines convolutional and self-attention layers. In addition, the use of relative positional embeddings is crucial for allowing the model to generalize to sequences of varying lengths. A similar trend is observed in fig. S7, where we retrained both eFold and UFold architectures on a set of RNA families, holding out one family for testing. When trained on families of short RNAs and tested on the long 16*S* rRNA family, UFold experienced a drastic drop in performance, whereas eFold maintained performance comparable to that observed on test sets of short RNAs. eFold architecture ablation studies revealed that removing either the self-attention (eFold blocks) or the pair-wise representation and communication (the CNN inside the eFold blocks) led to consistently lower performance across all test sets (Table 4 and fig. S8). These results underscore that both architectural components are important for maintaining prediction accuracy across RNA families of varying lengths and complexities.

**Table 4.** Ablation and baseline performance F1. Average F1 scores (0 to 1) for eFold, ablation 1 (CNN-only; no self-attention) and ablation 2 (self-attention–only with no information exchange between sequence and pair representations) across PDB, ArchiveII, Viral mRNA, and Long ncRNA test sets. The best model per test set (column) is in bold.

Dataset	PDB	ArchiveII	Viral mRNA	Long ncRNA
**Model**				
eFold	**0.89**	0.60	**0.73**	**0.44**
Ablation 1	0.79	0.56	0.64	0.40
Ablation 2	0.61	0.53	0.58	0.39
UFold	**0.89**	**0.61**	0.67	0.35

While all methods presented in this study were designed to predict Watson-Crick base pairs, deep learning models inherently avoid assumptions about base pair types and can learn less common interactions directly from data. We demonstrate this capability in table S2 and fig. S6, where all models were evaluated on two dedicated test sets: one containing noncanonical base pairs and the other pseudoknots. Classical methods failed to predict these structural features entirely, whereas deep learning approaches successfully identified both noncanonical pairs and pseudoknots, though with reduced accuracy compared with canonical base pair predictions. This performance gap likely stems from the limited availability of pseudoknot examples and noncanonical interactions in training datasets (table S3).

For an illustrative example, we also present a comparison of a functional element from the lncRNA polyadenylated nuclear RNA (PAN) structure model of Kaposi’s sarcoma herpesvirus (1.1 kb), as predicted by various state-of-the-art algorithms, against the reference structure derived from ex vivo deprotonated RNA probed with SHAPE (Fig. 5B) (*28*). We show both the pairing matrix and the resulting secondary structure figure. We see that UFold completely fails to predict this structure, while eFold and EternaFold both predict a good model with a F1 score of 0.86, outperforming RNAstructure Fold and MXFold2 F1 score of 0.65 and 0.55 respectively.

## DISCUSSION

In this study, we addressed two important challenges in the field of RNA secondary structure prediction: the limited generalizability of existing algorithms and the necessity for a robust, end-to-end predictive model. We highlighted the difficulty in predicting the structures of a broad class of biologically relevant RNA molecules, including mRNAs, viral RNAs, lncRNAs, and pri-miRNAs, with conventional algorithms. By curating a list of published structure models obtained through chemical probing, we demonstrated the shortcomings of ML-based approaches in generalizing to novel RNA types beyond their training sets. Our work was directed along two paths: first, the compilation of a new, diverse dataset of RNA structures; and second, the development of eFold, a cutting-edge model for RNA secondary structure prediction.

The dataset that we created encompasses secondary structures for 1456 human mRNA regions and 1098 pri-miRNAs, as determined through chemical probing. This collection broadens the spectrum of RNA structures available for research, addressing a vital deficiency in the current data landscape. The prevailing datasets, predominantly composed of short ncRNAs, have not sufficiently supported the development of algorithms capable of accurately predicting RNA structures. By integrating a wide range of RNA families and lengths, our objective was to enhance the predictive models’ ability to generalize.

The eFold architecture, drawing inspiration from the Evoformer block of AlphaFold and incorporating elements of traditional architectures, marks a notable advancement in the prediction of RNA secondary structures. This end-to-end deep learning model was trained using the RNAndria database and more than 300,000 secondary structures from diverse sources. Our evaluations reveal that eFold outperforms existing state-of-the-art end-to-end methods, especially in predicting complex structures, such as those of lncRNAs. This achievement highlights the critical importance of diversifying the training dataset in terms of structure diversity, length, and complexity to augment the precision of ML-based models. We intend to substantially scale up the eFold method in future work, both in terms of dataset and model size, as our current model is relatively small (1.5 million parameters) compared with other work (8.6 million for UFold and 32 million for RNAformer). A larger model could allow to better learn from larger dataset, as we collect more data for RNAndria database.

This research underlines the pressing need for datasets that encompass a broad array of long RNA sequences and complex structures. At present, the accuracy of predictions for a curated selection of published lncRNA structures ranges from 40 to 50%. This stands in stark contrast to the 90 to 100% accuracy rates achieved for short ncRNAs within the PDB. The introduction of the eFold model and the RNAndria database represents an important stride toward mitigating this disparity. With the inclusion of a larger and more varied dataset, potentially encompassing alternative structures, achieving a level of predictive precision for long RNAs comparable to the high accuracy rates currently observed for short ncRNAs becomes a tangible goal. Such precision is essential for unraveling the complex roles and functions of RNA structures in biological systems.

Moreover, this work highlights inherent limitations in training and predicting alternative RNA structures, primarily due to the scarcity of experimentally verified alternative conformations. As a result, our focus was on structures most likely to adopt a single predominant conformation, as indicated by high AUROC scores. We also chose not to prioritize the prediction of noncanonical base pairs or pseudoknots, although our models demonstrate the capacity to predict some of these features without explicit design. The reduced accuracy in these cases is likely attributable to the limited number of available examples of noncanonical interactions and pseudoknot structures in current datasets. Moving forward, we plan to systematically collect and incorporate more diverse and well-annotated examples of alternative conformations, noncanonical base pairs, and pseudoknots, thereby enhancing the scope and accuracy of future versions of our models.

## METHODS

### Library design

To generate the human mRNA library, all human protein-coding transcripts were downloaded from GENCODE Release 43 (*29*). Every transcript with a non-AUG start codon or without an annotated 5′ untranslated region, coding sequence, and 3′ untranslated region (3′UTR) was discarded. To focus on 3′UTRs and maximize the likelihood of successful reverse transcription polymerase chain reaction (PCRs), for each gene, the longest common suffix (i.e., subsequence beginning at the 3′ end) among all isoforms was selected. Each gene was discarded if it had no annotated name, if its longest common suffix was shorter than 253 nt, or (to minimize mispriming and misalignment) if its longest common suffix contained any 41-mers that were present in the longest common suffix of any other gene. To verify that alignment to the wrong gene would be minimal, a simulated FASTQ file containing every 128-nt segment in 64-nt increments from every gene’s longest common suffix was aligned to all longest common suffixes with Bowtie2 v2.5.1 (*30*).

Primers for human mRNAs were designed against the longest common suffixes of all remaining genes using Primer3 v2.6.1 (*31*) with the parameters PRIMER_TASK = generic, PRIMER_PICK_LEFT_PRIMER = 1, PRIMER_PICK_INTERNAL_OLIGO = 0, PRIMER_PICK_RIGHT_PRIMER = 1, PRIMER_PRODUCT_SIZE_RANGE = 253-1021, PRIMER_OPT_SIZE = 28, PRIMER_MIN_SIZE = 20, PRIMER_MAX_SIZE = 36, PRIMER_WT_SIZE_LT = 0.0, PRIMER_WT_SIZE_GT = 0.0, PRIMER_MIN_TM = 65.0, PRIMER_OPT_TM = 75.0, PRIMER_MAX_TM = 80.0, PRIMER_PAIR_MAX_DIFF_TM = 10.0, PRIMER_GC_CLAMP = 0, PRIMER_MAX_NS_ACCEPTED = 0, P3_FILE_FLAG = 0, PRIMER_EXPLAIN_FLAG = 1, SEQUENCE_OVERHANG_LEFT = TAATACGACTCACTATAGGG. A T7 promoter ending in three G bases was prepended to every forward primer. Genes that received no primer pairs were discarded; for those that received more than one pair, the pair that would produce the longest amplicon was selected. The sequences of the amplicons (including only the last three Gs of the T7 promoter) were used as the reference sequences in the subsequent analysis.

To select amplicons for verification in human embryonic kidney (HEK) 293 T cells, four microarray datasets of gene expression in HEK 293 T cells from two studies (*32*) were downloaded from the National Center for Biotechnology Information’s Gene Expression Omnibus (*33*) (accession nos. GSM389756, GSM389757, GSM609202, and GSM609205). Microarray probe IDs were converted to gene names using DAVID (*34*). The amplicons of at most 600 nt (the maximum paired-end read length on Illumina sequencers) were ranked by the minimum expression level of their genes among the four datasets (to be conservative); the 96 highest-ranked amplicons were chosen for mutational profiling in HEK 293 T cells.

To generate the pri-miRNA library, human genome annotations were downloaded from miRBase (*35*). After removing redundant sequences, 1292 miRBase hairpins were selected for downstream library creation. The miRBase hairpins were then padded with their flanking genomic sequence to a total of 192 nt. These 1292 sequences were then separated into three sublibraries to reduce jackpotting during library processing. To each construct, a sublibrary-specific pair of 19-nt universal primer binding regions were added to minimize primer-associated PCR and reverse transcription bias, bringing the full-length library size to 230 nt. The final sequences were then synthesized as an oligo pool (Agilent Technologies).

### DMS data generation

For the in vitro transcription of the human mRNA library, all the designed amplicons were amplified via PCR, adding a T7 promoter sequence at the forward primer (5′ TAATACGACTCACTATAG 3′) using a 2X PCR PreMix (Syd Labs, catalog no. MB067-EQ2N), human cDNA (ZYAGEN, catalog no. HD-UR-40), and an INTEGRA VIAFLO384 channel pipette. For each 384-well plate, 5 μl of each individual reaction were pooled together and purified using a Zymo DNA cleanup kit (catalog no. D4003). The PCR product pool was used as template for the in vitro transcription, using the T7 MEGAscript kit (Thermo Fisher Scientific, catalog no. AMB13345) according to the manufacturer’s instructions. After deoxyribonuclease (DNase) treatment, the transcribed RNAs were purified using a Zymo RNA cleanup kit (catalog no. R1017). The purified RNAs (10 μg) were diluted in 10 μl of nuclease-free water, denatured for 1 min at 95°, and placed on ice for 2 min. Refolding buffer (88.5 μl) (sodium cacodylate, 397.5 mM and MgCl_2_, 6 mM final) was added, and the RNAs were left refolding at 20 min at 37°C. Then, 1.5 μl of DMS was added to obtain a 1.5% final concentration, for 5 min at 37°C, while shaking at 600 RPM. The reaction was stopped by adding 60 μl of β-mercaptoethanol, followed by Zymo RNA cleanup. Libraries were generated as previously described (*36*), and the indexed samples were sequenced in an Illumina NextSeq 2000.

For the pri-miRNA library, in vitro transcription templates were prepared via 8 cycles of PCR using 2× Q5 Master Mix (New England Biolabs, catalog no. M0492S) with a T7 promoter containing forward primer as previously described. Following column cleanup (Zymo Research, catalog no. D4013), 100 ng of template DNA was used in a 2-hour in vitro transcription reaction using the HiScribe T7 High Yield RNA (New England Biolabs, catalog no. E2040S) kit according to the manufacturer’s instructions. Following DNase digestion (Invitrogen, catalog no. AM2238) and column cleanup (Zymo Research, catalog no. R1017), 1 ug of RNA from each library was DMS treated separately as described above, substituting a 1.5% final DMS concentration for 2.5%. Following DMS treatment, the samples were reverse transcribed with Induro Reverse Transcriptase (New England Biolabs, catalog no. M0681L) for 30 min at 55°C using the appropriate reverse primer for each library according to the manufacturer’s protocol. After alkaline hydrolysis with 1 μl of 4 M NaOH, cDNA was cleaned and concentrated using Zymo Oligo Clean and Concentrator (Zymo Research, D4060). Sublibrary-specific primers were then used to amplify 1 μl of cDNA template for 24 cycles using Advantage 2 PCR Mix (Takara Bio, catalog no. 639206). Last, library indexing was carried out with the NEBNext Ultra II DNA Library Prep Kit for Illumina (New England Biolabs, catalog no. E7645S) and sequenced on a NextSeq 1000 P2 cartridge (Illumina, catalog no. 20046813) in accordance with the manufacturer’s instructions.

### DMS data processing and filtering

For the processing of raw reads, the fastq files were handled using SEISMIC-RNA (*37*), which aligned the reads to the reference sequence and quantified mutations per base. Sequences with less than 3000 aligned reads or where less than 50% of bases had coverage of at least 3000 reads were excluded. In some cases, an untreated RNA sample was sequenced to provide a baseline, aiding in the identification of abnormally high mutation rates (>0.3) that might indicate inaccuracies in the reference sequence. If mutations consistently occurred toward a specific base, then the reference sequence was updated; otherwise, the sequence was discarded. Biological replicates were analyzed to check experimental consistency. Replicates with a Pearson correlation below 0.8 were removed, while those with higher correlation were averaged for further use. Bases were masked with a value of −1000 if their coverage was below 3000, if they were G or U bases, or if they were part of the primer region in the pri-miRNA dataset. Signal normalization was then performed per reference, using the 95th percentile as the maximum value, with values above this threshold clipped to 1. The RNAstructure Fold algorithm, incorporating the DMS signal as a constraint, was then applied. Structures with an AUROC of less than 0.8 in relation to the DMS signal were excluded. Through these measures, 15% of the pri-miRNA dataset and 70% of the human mRNA dataset were removed, ensuring the high quality of the remaining data.

### Data quality assessment

Pri-miRNA has replicates for 99% of the references, and human mRNA has replicates for 50% of the references. This is because we have very stringent filtering steps, so some references were valid in one experiment and not in another. We estimated the reproducibility of our data by comparing our replicates. The DMS signal replicates were compared using the Pearson correlation score. The median Pearson score was 1.0 for pri-miRNA and 0.94 for mRNA. Note that in the final dataset, both replicates were aggregated into a single signal.

We also estimated the robustness of the data as the number of reads decreases. The DMS-MaPseq method consists in counting the mutated bases at each position across a large number of reads. To estimate the error due to the number of reads, we model the mutation fraction as a binomial distribution divided by the number of trials. We bootstrap 10 DMS signals for each reference from the original signal using this model. We predict the structure using RNAstructure and these DMS signals. The structures are systematically the same when predicted with the original signal and the bootstrapped signals, suggesting that subsampling error does not have an influence on the structure prediction.

### Test sets

For our test sets, we sourced data from a variety of published papers by different research groups. In selecting entries from the PDB, we focused on those classified under “Polymer Composition” as RNA and with a “Number of Assemblies”, “Number of Distinct Molecular Entities,” and “Total Number of Polymer Instances” all set at 1. This approach yielded 355 entries, which were then converted from tertiary to secondary structures using the RNApdbee webserver, applying the default settings: 3DNA/DSSR as the conversion software and the hybrid algorithm method.

The ArchiveII dataset was downloaded from Mathew lab website (https://rna.urmc.rochester.edu/publications.html). We only removed 5 sequences that were longer than 2000 nt, resulting in a test set with 3370 sequences.

The viral structure dataset was created by segmenting long viral RNA sequences into smaller, independent modules. We used the HIV structure from Watts *et al.* (*38*), the severe acute respiratory syndrome coronavirus structure from Lan *et al.* (*26*), the hepatitis virus structure from Mauger *et al.* (*39*), and the Alphavirus structure from Kutchko *et al.* (*40*). For each of these structures, we identified modular structures characterized by fully closed loops and high agreement between the structure and chemical probing data (AUROC >0.8). After segmenting the chemical probing signal (DMS or SHAPE), RNAstructure was rerun with each fragment. Fragments were retained if the new structure aligned with the corresponding segment of the chemical probing signal (AUROC > 0.8 and F1 > 0.8).

The lncRNA dataset was sourced from Bugnon *et al.* (*11*), with the only modification being to cut sequences exceeding 2000 nt in length, using the same method as for the viral structures. We did not use the last filtering step of the cutting process as we found that it made almost no difference in the test results of all algorithms and models. To generate a sufficiently large dataset, we implemented a segmentation process that divided RNA sequences into smaller substructures. This step was crucial for expanding our training data. However, this segmentation was not necessary for the ArchiveII dataset, which already contained an ample number of diverse sequences, providing a comprehensive representation of RNA structures without further subdivision.

### External database curation

We combined the public databases bpRNA (*13*) and Ribonanza (*17*) into a pretraining dataset. We applied the following filtering steps: First, we removed duplicate sequences within the databases. We only kept sequences with the canonical bases ACGU. The T bases were converted to U. We filtered out the sequences below 10 nt. We removed sequences for which we have a sequence but no structure. We removed sequences that are common between datasets. For Ribonanza specifically, we applied the following filtering: We filtered low reads and low signal-to-noise ratio (S/N ratio) data. The cutoff was set to more than 500 reads and a S/N ratio—a quality indicator provided by the Ribonanza dataset—greater than 1. The data include structures predicted with EternaFold. To ensure that the EternaFold-predicted structure was matching the signal, we computed the AUROC between the structure and one of the chemical probing signals for each sequence. We used DMS by default and SHAPE if DMS was filtered out. If the AUROC was below 0.8, then the structure was filtered out. We removed redundant sequences within the dataset using BLAST. The primers were masked. If two sequences had over 80% matches on a sequence of more than 112 nt, we kept only the best covered sequence. Note that 112 nt correspond to 80% of the most represented length minus the primers.

The last dataset used for pretraining is a synthetic dataset. We gathered sequences from RNACentral by sampling uniformly from all RNA clans, then added a balanced proportion of mRNA and viral sequences, and lastly predicted the structure of each sequence using RNAstructure Fold. We combined all training data and removed any sequence similar to the test sets with a BLAST analysis using the same parameters as before. The final dataset called “efold_train” contains 306,557 sequences up to 1024 in length.

### Structure visualization

All the secondary structures available in RNAndria, along with their DMS reactivity per base, have been plotted from the command line using the tool RNArtistCore (https://github.com/fjossinet/RNArtistCore). Built with Kotlin (https://kotlinlang.org/), RNArtistCore follows a heuristic approach to identify a nonoverlapping two-dimensional (2D) layout for an RNA secondary structure.

At first, RNArtistCore computes the structural domains (helices and junctions) from paired positions. In 3D, junctions connect and orient helices to produce the biologically relevant 3D architectures. Each junction is associated to a class depending on the number of helices connected (apical loops, inner loops, three-way junctions, four-way junctions, etc.). Starting from the 5′ end, RNArtistCore is processing each junction to find orientations for its helices to avoid overlapping between upstream and downstream structural domains in a 2D sketch.

Except for apical loops, the drawing engine implemented in RNArtistCore splits the connected helices in a junction between a single ingoing helix and outgoing ones. The modeling of RNA concepts of RNArtistCore links each junction class to a default positioning for its outgoing helices, relatively to the orientation of its ingoing one. If the default position for an outgoing helix produces an overlap between the next junction and at least one upstream structural domains, then RNArtistCore searches for a new position among those still available. When the orientation of an outgoing helix has been defined, this helix becomes the ingoing one for the next junction to be processed.

Once a nonoverlapping layout has been found, it is used to produce an SVG drawing according to some user-defined instructions. RNArtistCore provides a simple and expressive syntax (also known as Domain Specific Language) to allow the user to parameter the final result. The instructions are described in a script whose name and location has to be specified on the command line. Among all the options available (detail levels, colors, line widths, etc.), one can easily link a dataset (like DMS reactivities) to the structure and map its values to a color gradient.

Some 2D RNA structures can contain nested junctions for which RNArtistCore could not be able to find any nonoverlapping organization. In such cases, RNArtistCore can be configured to go backwards to test different layouts for upstream junctions that could unlock the nested ones. For this study, we allowed RNArtistCore to go back until 10 previous junctions if necessary.

### Models architecture

Model inputs are RNA sequences only. The output is a 2D RNA structure prediction in the form of a pairing matrix. The sequence is one-hot encoded and then fed through a learned embedding layer (dimension = 64). This learned representation is the input to the eFold module. The first step in this module is constructing the pair representation. The one-hot encoded sequence is transformed using a Kronecker outer product (same as Ufold) to create an LxLx17 pair representation. The sequence representation goes through a relative positional multihead self-attention module with bias from the paired representation. This module consists of a multiheaded attention mechanism with learned relative position encodings (*41*) and pair representation bias added to the logits before the softmax function. The sequence representation goes through a feed-forward layer followed by a 1D convolution module. This includes pointwise convolutions followed by depthwise convolution (number of output channels = 128) and lastly another pointwise convolution (number of output channels = 64). This is followed by another feed-forward layer and a two-layer deep sequence representation transition multilayer perceptron (MLP). There are skip connections between each module. The output of the transition MLP is transformed using an outer mean product and added to the pair representation for the second trunk of the eFold module. The pair representation is sent through a dilated 2D ResNet with two residual blocks of depth. The output is followed by a two-layer deep pair representation transition MLP. The eFold module is repeated four times before the output layers. Pairing matrix predictions are sent through a final 2D residual block head (output dimensions = LxLx1). Loss is calculated with binary cross entropy. The pairing matrix is postprocessed by enforcing constraints such as no sharp loops and only one nucleotide can be paired with another. We reuse the same optimization method as UFold to find the pairing matrix respecting the constraints with minimum modification of the model output. A fixed threshold of 0.5 is used to call base pairs. Because the resulting probabilities are almost always near 0 or 1, applying a 0.5 threshold to call base pairs yields consistent results, and the specific choice of threshold has minimal impact on pair accuracy.

### Training and evaluation

The model is developed using the Pytorch and Lightning libraries. We use the binary cross entropy loss for structure prediction. We use a cluster of 8 Nvidia RTX4090 and train all models and experiments using the Distributed Data Parallel (DDP) strategy for 15 epoch. Because the results can vary from epoch to epoch, we average the weights of the last five epochs (epochs 10 to 15) to get the final weights used in the experiments. Because of large memory constraints, we run the model with a batch size of 1 and thus do not require any padding. Using gradient accumulation, the effective batch size is 256. We train with Adam optimizer with a learning rate of 0.0003.

The model was trained on sequences up to 1024 nt, but it can be applied to sequences of any length, with the primary limitation being the available graphics processing unit (GPU) or central processing unit memory. More advanced strategies, such as model parallelization across multiple devices, could enable prediction on substantially longer sequences.

The test sets are run separately on one GPU to ensure maximum precision in the metric computations. All the code is freely available on Github at https://github.com/rouskinlab/efold and the datasets on Huggingface at https://huggingface.co/rouskinlab.

### Ablation experiments

To assess architectural contributions, we performed two ablation experiments (ablation 1: CNN-only; ablation 2: self-attention–only/no-exchange). For ablation 1 (CNN-only), we removed all eFold blocks, thereby eliminating self-attention and within-block exchange, and pass the Kronecker-constructed pair representation directly to the unchanged decoder (see fig. S8A). For ablation 2 (self-attention–only/no-exchange), we removed the pairwise CNNs in every eFold block, which also removes the pair-bias feedback from the pair representation to sequence representation. Last, a single-pair representation was derived from the sequence features on the last block and passed to the same decoder (see fig. S8B). In performing this ablation study, some parameters were removed in both ablations. eFold has 1,493,420 parameters, ablation 1 has 595,100 parameters, ablation 2 has 992,720 parameters. All ablation variants used identical datasets, preprocessing, loss functions, and training protocols as the main model, with identical decoder architecture maintained throughout.
