# Supplementary material for: Diverse database and machine learning model to narrow the generalization gap in RNA structure prediction
Source: Sci Adv. 2026 Feb 25;12(9):eadz4967. doi: 10.1126/sciadv.adz4967 (PMC12935039; doi:10.1126/sciadv.adz4967)
Supplement: Supplementary file 1 — Figs. S1 to S8 Tables S1 to S3 [file sciadv.adz4967_sm.pdf]

Science Advances
AAAS

Supplementary Materials for

Diverse database and machine learning model to narrow the generalization gap in RNA structure prediction

Albéric A. de Lajarte *et al.*

Corresponding author: Silvi Rouskin, silvi@hms.harvard.edu

Sci. Adv. **12**, eadz4967 (2026)
DOI: 10.1126/sciadv.adz4967

This PDF file includes:

Figs. S1 to S8
Tables S1 to S3

Supplementary Figures (S1–S8)

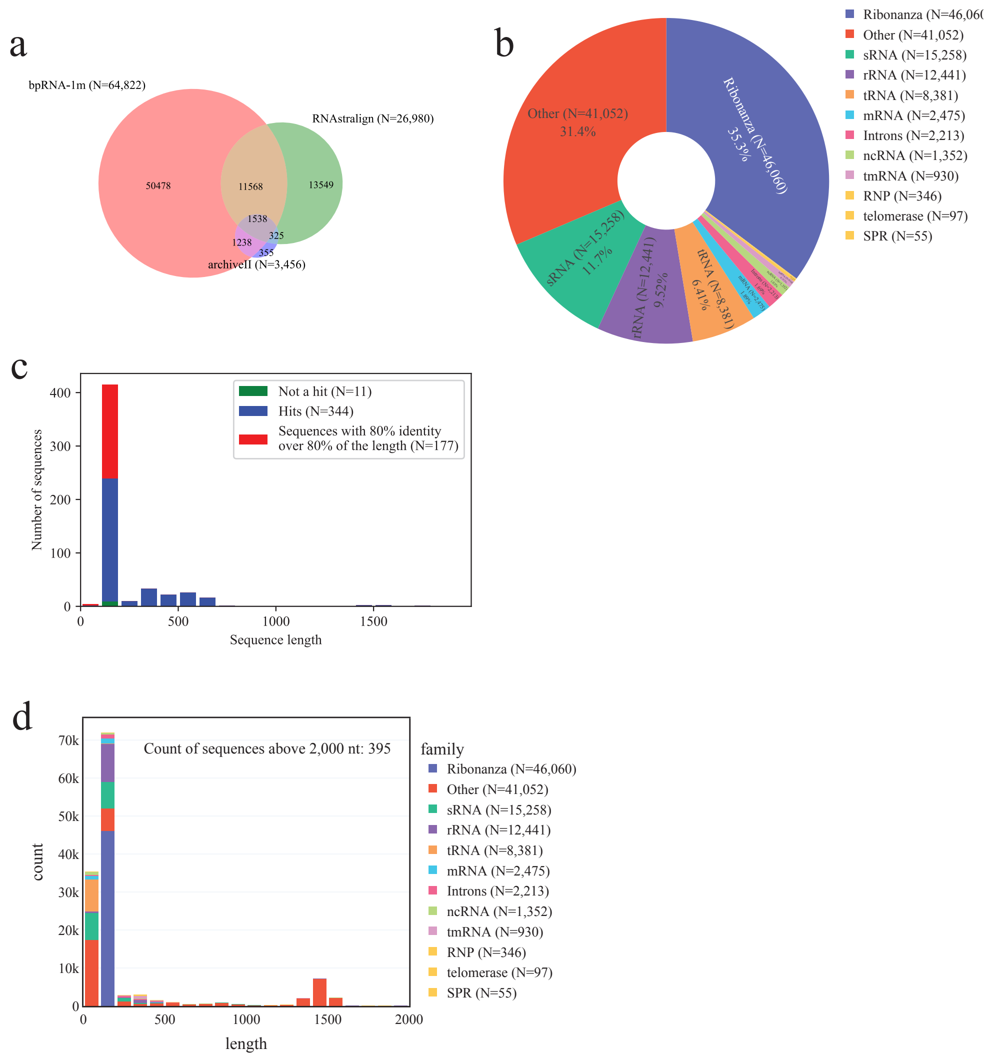

Supplementary Figure 1. Representation of the external databases and of the families and lengths of the pre-train set.
(a) The common sequences between bpRNA, RNAstralign and ArchiveII are represented using a Venn diagram.
(b) The distribution in families of the compiled train set, displayed as a pie chart. Ribonanza counts as a family since we don't have labels for it.
(c) BLAST analysis of the ArchiveII test set compared to bpRNA and RNAstralign, after removing common sequences.
(d) The distribution of the length of the compiled train set, displayed as a histogram.

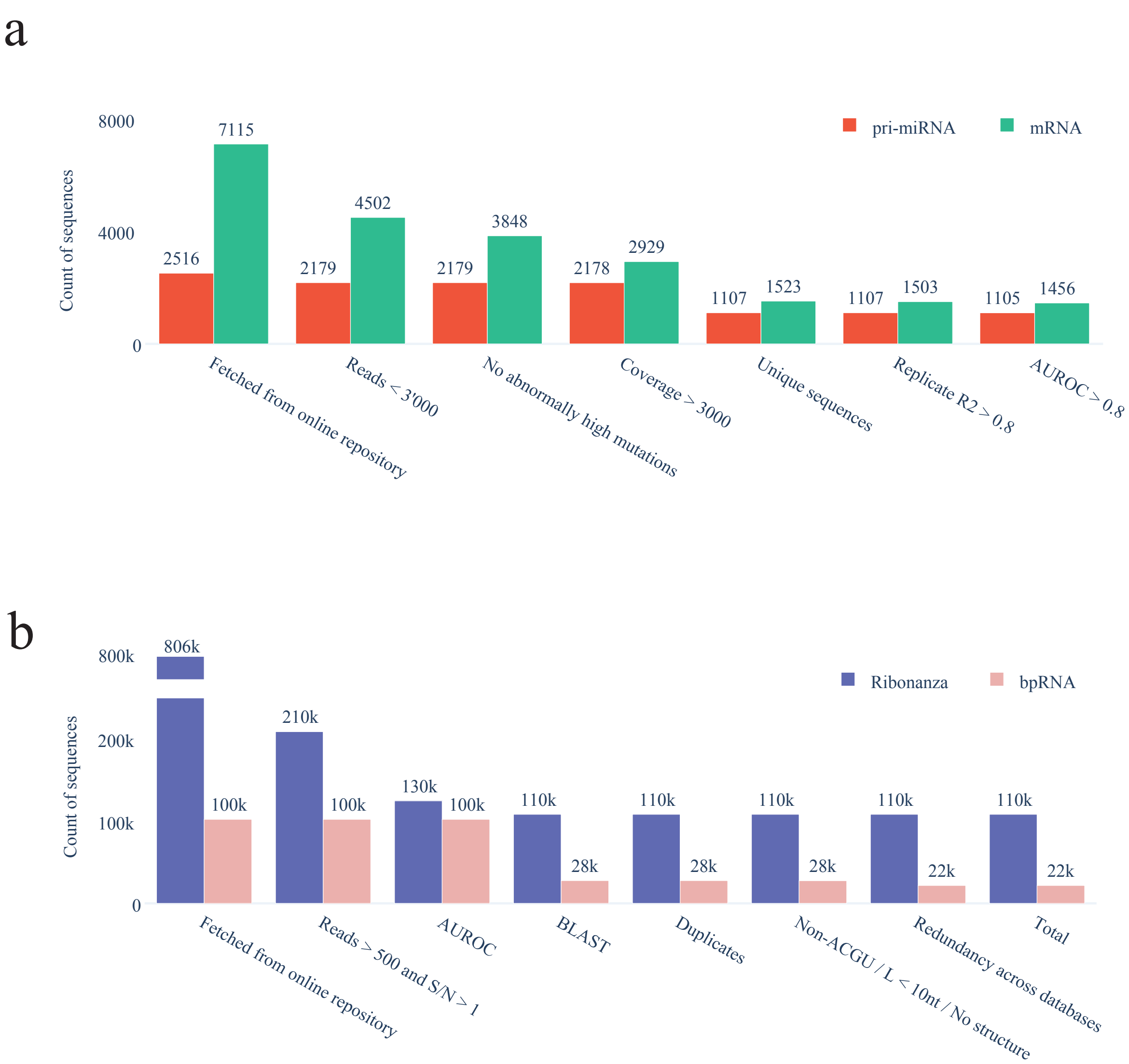

Supplementary Figure 2. Filtering steps of the datasets.

(a) Dataset size at each filtering step for the pri-miRNA (red) and mRNA (green) datasets before fine-tuning.

(b) Dataset size at each filtering step of the bpRNA (pink) and Ribonanza (blue) datasets before pretraining. Note that the y axis is discontinuous for clarity.

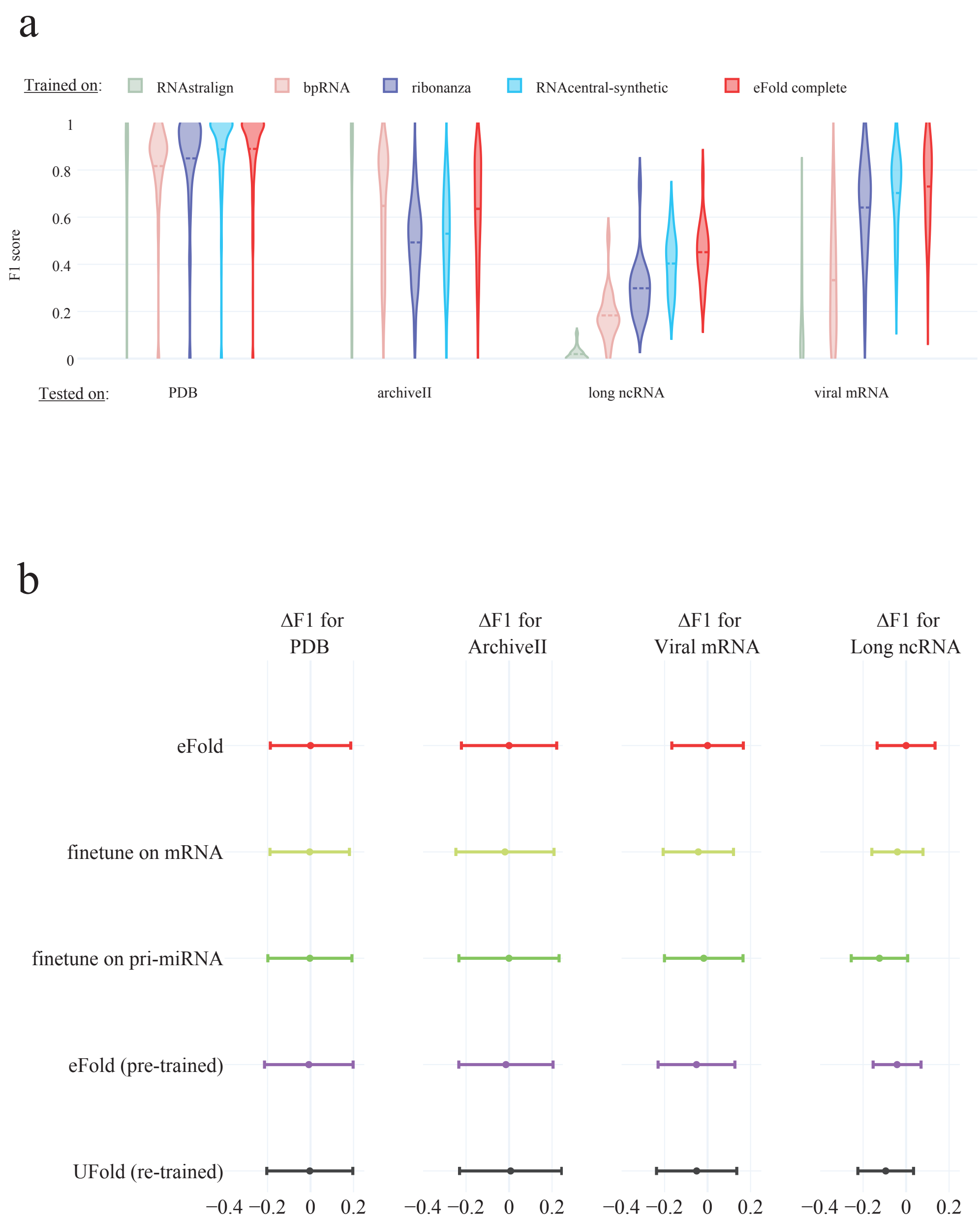

Supplementary Figure 3. Ablation study.
(a) F1 distribution on each test set when training the eFold architecture on each database individually. The final eFold model, trained on bpRNA (pink), Ribonanza (blue), and synthetic dataset (light blue) is plotted for reference in red.
(b) Ablation study for different architecture and training strategies. The baseline model in red is eFold (pre-trained then fine-tuned). We try fine-tuning on only the mRNA (light green), only the pri-miRNA (dark green), only pretraining (no fine-tuning, purple), and complete pre-training and fine-tuning with the UFold architecture (re-training, black). The delta F1 score is with respect to the baseline mean F1 score per dataset. The error bar corresponds to one standard deviation.

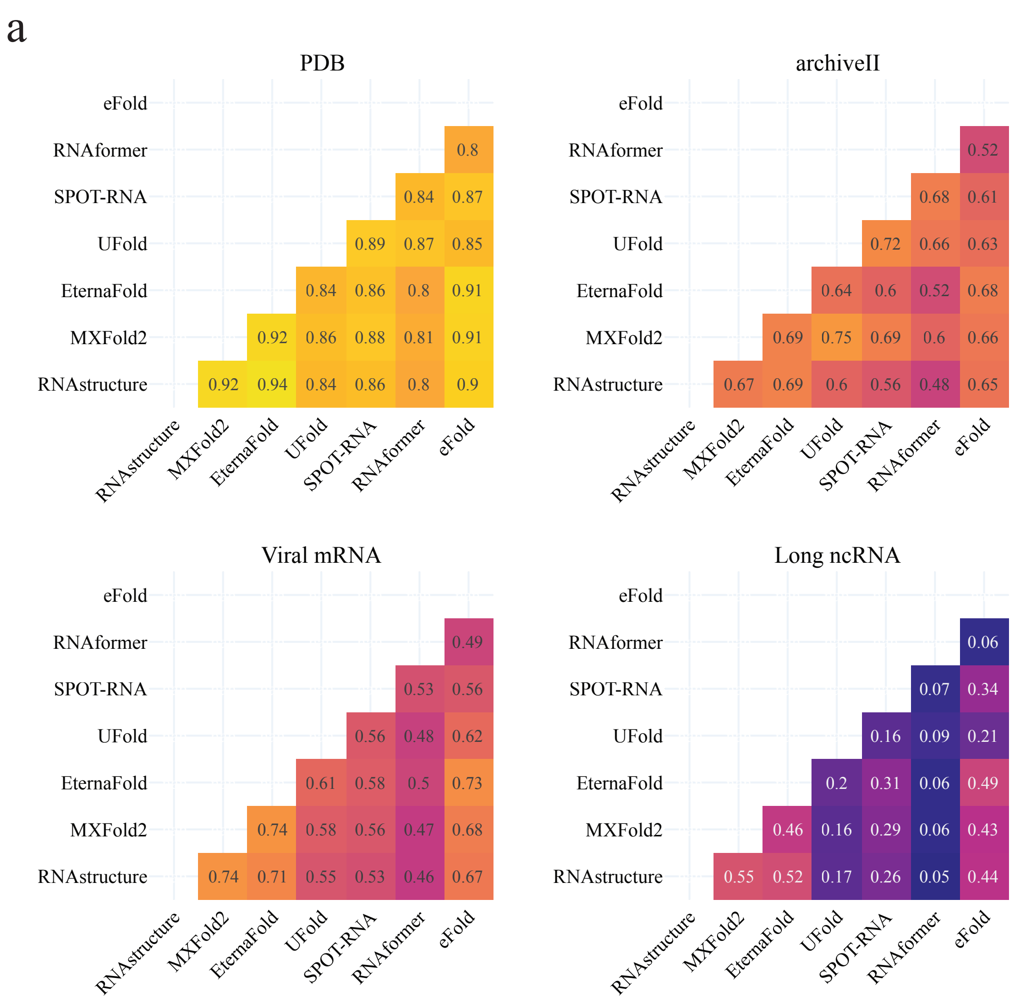

Supplementary Figure 4. Prediction similarity.

(a) Similarity matrix between prediction of different algorithms, on the four test sets. Each algorithm's prediction is compared to each other algorithm, and the average F1 score between predictions is reported.

a

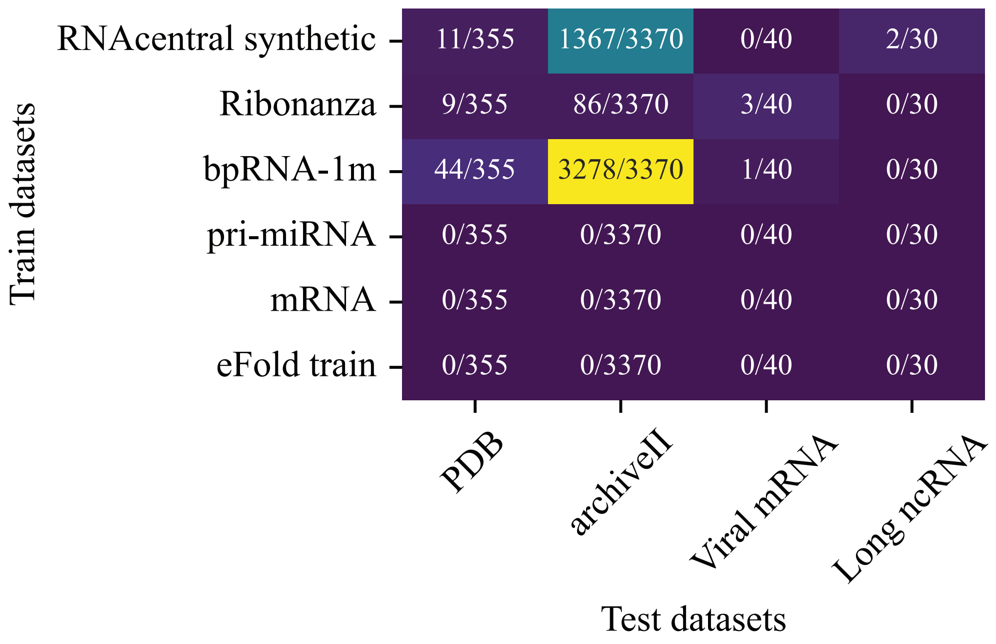

Supplementary Figure 5. Train and test sets similarity.
(a) Number of sequences from the training set that match the corresponding testing set. A match is found using BLAST, with a threshold at 60% of matches on at least 60% of the sequence.

a

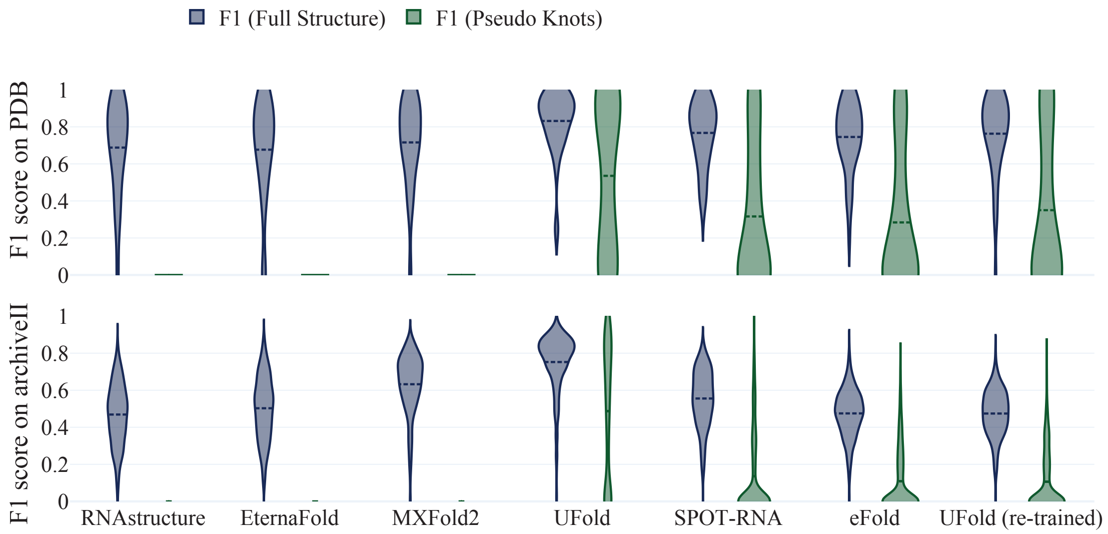

Supplementary Figure 6. Pseudoknots prediction performance.

(a) Distribution of F1 score on structures of PDB (first row) and archiveII (second row) containing pseudoknots. Total number of pseudoknots is 43 in PDB and 974 in ArchiveII. In blue is shown the F1 score over the whole structure, and in green over the pseudoknots base pairs.

a

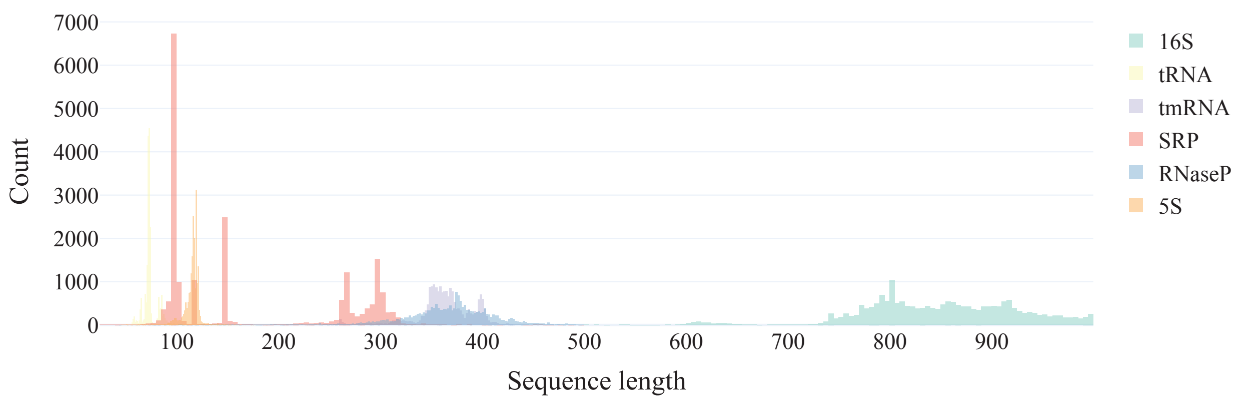

b

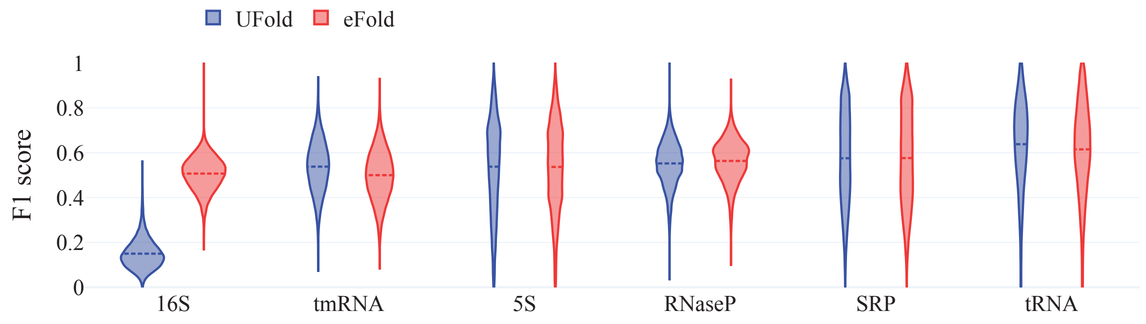

Supplementary Figure 7. Family generalization test.

(a) Distribution of F1 score for the eFold and UFold architecture trained from scratch on 6 families from RNAcentral, with synthetic structure predicted by RNAstructure Fold. Note RNAstructure Fold achieves mean F1 of 0.9 for PDB families, and these families are a part of the PDB database. Each violin plot is the result of a complete training on all families except one left aside for testing. Each family set contains 20k sequences; as training is done on 5 out of 6 families, 100k sequences are used for training and 20k sequences are used for testing.

(b) Sequence length distribution of the family types used for training and testing.

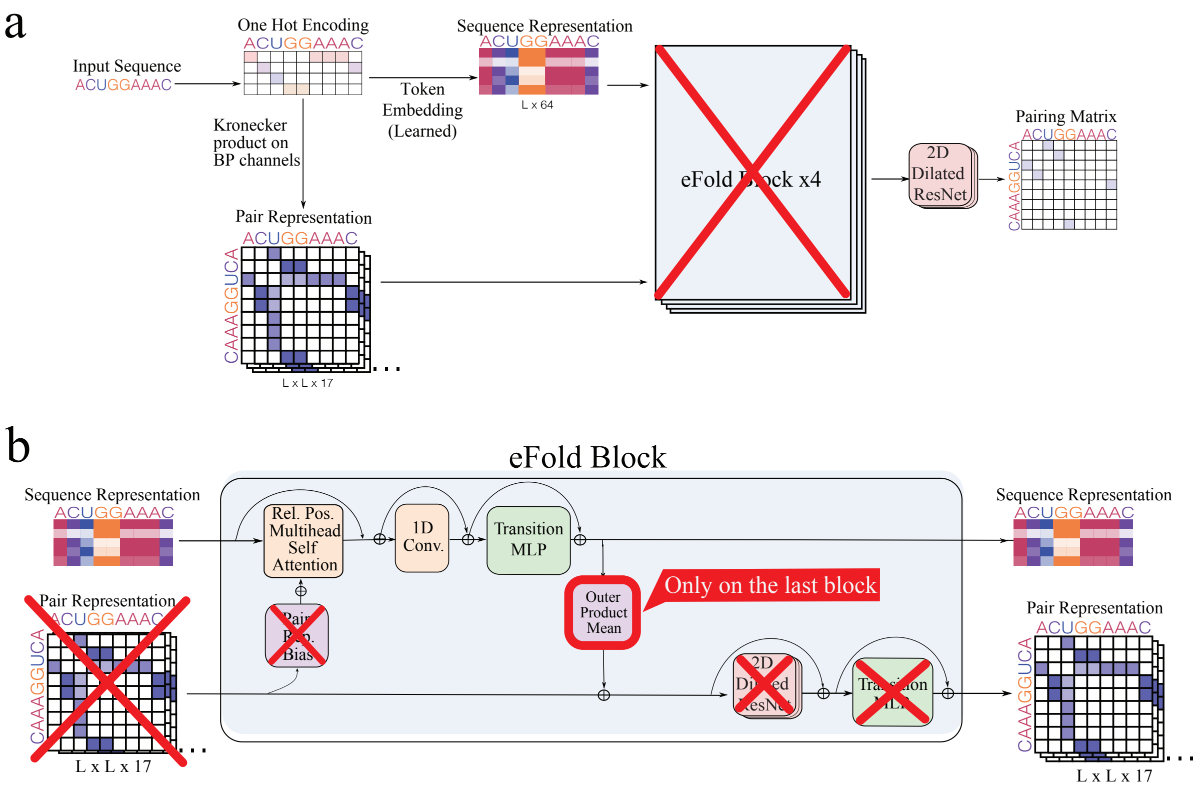

Supplementary Figure 8. Architectural ablations of eFold.

(a) Ablation 1 — CNN-only. All eFold blocks (and thus all attention operations and the sequence stream) are removed; the Kronecker-constructed pair representation is fed directly to the unchanged 2D dilated ResNet decoder, yielding a minimal CNN-only baseline.

(b) Ablation 2 — Self-attention-only with no information exchange. The within-block pairwise CNN and pair-to-sequence bias are removed, so attention operates without pair information; to produce a 2D output, a single pair representation is formed from the sequence features only on the last block and passed to the same decoder. No feedback occurs between pair and sequence streams at any point. The schematic mirrors Fig. 4, with crossed-out components indicating removals.

Supplementary Tables (ST1–ST3)

dataset	PDB				archiveII				Viral mRNA				Long ncRNA			
	Precision	Recall	F1	MCC	Precision	Recall	F1	MCC	Precision	Recall	F1	MCC	Precision	Recall	F1	MCC
**model**																
**RNAstructure Fold**	0.90	0.91	0.89	0.89	0.55	0.60	0.57	0.57	0.69	0.74	0.71	0.71	**0.46**	**0.52**	**0.49**	**0.49**
**EternaFold**	0.90	0.91	0.88	0.88	0.57	0.64	0.60	0.60	**0.75**	**0.81**	**0.77**	**0.78**	0.45	0.47	0.46	0.46
**MXFold2**	0.91	0.93	**0.90**	**0.91**	0.73	0.76	0.74	0.74	0.70	0.72	0.71	0.71	0.41	0.43	0.42	0.42
**UFold**	0.81	**0.97**	0.87	0.87	0.83	**0.89**	**0.85**	**0.85**	0.58	0.59	0.58	0.58	0.22	0.14	0.16	0.17
**E2EFold**	0.21	0.10	0.13	0.13	0.29	0.21	0.24	0.24	0.04	0.03	0.03	0.03	0.03	0.03	0.03	0.03
**SPOT-RNA**	0.85	0.92	0.87	0.87	0.69	0.73	0.70	0.70	0.68	0.50	0.56	0.58	0.34	0.21	0.26	0.27
**CNNFold**	**0.92**	0.60	0.65	0.67	**0.91**	0.43	0.52	0.57	0.56	0.04	0.07	0.12	0.39	0.01	0.02	0.07
**NeuralFold**	0.81	0.84	0.81	0.81	0.72	0.72	0.72	0.72	0.26	0.24	0.25	0.25	0.15	0.13	0.14	0.14
**RNAformer**	0.74	0.96	0.82	0.82	0.54	0.84	0.65	0.67	0.48	0.50	0.48	0.48	0.27	0.08	0.07	0.07
**eFold**	0.89	0.93	0.89	0.89	0.57	0.64	0.60	0.60	0.70	0.75	0.73	0.73	0.46	0.43	0.44	0.44

Supplementary Table 1: Performance of all algorithms on the test sets. Average Precision, Recall, F1 and MCC score between the predicted structure model of each algorithm against a published structure model across four distinct test sets: PDB, ArchiveII, Viral mRNAs, and Long ncRNAs. The cells are colored, with yellow corresponding to the maximum F1 score of 1, and dark blue to the minimum F1 Score of 0. The best model per test (column) is in bold.

dataset	RNAstralign non-canonical		
	Precision non-canonical	Recall non-canonical	F1 non-canonical
model			
EternaFold	0	0	0
MXFold2	0	0	0
RNAstructure	0	0	0
SPOT-RNA	0.11	0.06	0.08
UFold	0.16	**0.26**	**0.18**
eFold	**0.80**	0.01	0.01
uFold (retrained)	0	0	0

Supplementary Table 2: Performance on non-canonical base pairs. Average Precision, Recall, and F1 score over the non-canonical base pairs of the RNAstralign dataset. The precision is the ratio of predicted non-canonical that are true base pairs, the recall is the ratio of true non-canonical that are correctly predicted as base pairs, and the F1 score is the harmonic mean of precision and recall. See Supplementary Table 3 for the numbers of each base-pair type.

Pretraining		
**Pairs**	**Percentage**	**Count**
**Canonical Pairs**	**98.68**	**13733057**
A-U	38.44	5350389
C-G	49.76	6925480
G-U	10.47	1457188
**Non Canonical Pairs**	**1.32**	**184374**
A-A	0.1	13631
A-C	0.26	36669
A-G	0.43	60532
C-C	0.07	9876
C-U	0.16	21814
G-G	0.12	17023
U-U	0.18	24829

Finetuning: pri_mRNA & human_mRNA		
**Pairs**	**Percentage**	**Count**
**Canonical Pairs**	**100**	**323878**
A-U	27.32	88470
C-G	60.85	197072
G-U	11.84	38336
**Non Canonical Pairs**	**0**	**0**

RNAStralign		
**Pairs**	**Percentage**	**Count**
**Canonical Pairs**	**95.04**	**4282796**
A-U	23.22	1046363
C-G	58.41	2632263
G-U	13.41	604170
**Non Canonical Pairs**	**4.96**	**223710**
A-A	0.26	11542
A-C	0.85	38385
A-G	2.28	102660
C-C	0.13	5794
C-U	0.26	11901
G-G	0.42	19058
U-U	0.76	34370

Supplementary Table 3: Base-pair composition across training and RNAstralign datasets. The table reports the percentage and absolute count of base pairs broken down into canonical (A–U, C–G, and wobble G–U) and non-canonical (A–A, A–C, A–G, C–C, C–U, G–G, U–U) categories. Values are aggregated over all structures in each set.
